# Body composition differences in patients with Metabolic Dysfunction-Associated Steatotic Liver Disease

**DOI:** 10.3389/fnut.2024.1490277

**Published:** 2024-11-05

**Authors:** Karen D. Bernal-Contreras, Montserrat Berrospe-Alfaro, Regina López de Cárdenas-Rojo, Martha H. Ramos-Ostos, Misael Uribe, Iván López-Méndez, Eva Juárez-Hernández

**Affiliations:** ^1^Facultad de Ciencias de la Salud, Universidad Anáhuac, Huixquilucan, Mexico; ^2^Translational Research Unit, Medica Sur Clinic and Foundation, Mexico City, Mexico; ^3^Integral Diagnosis and Treatment Unit, Medica Sur Clinic and Foundation, Mexico City, Mexico; ^4^Gastroenterology and Obesity Unit, Medica Sur Clinic and Foundation, Mexico City, Mexico; ^5^Hepatology and Transplants Unit, Medica Sur Clinic and Foundation, Mexico City, Mexico

**Keywords:** steatosis, liver, body composition, obesity, muscle mass

## Abstract

**Background:**

Although body composition (BC) has been associated with Metabolic Dysfunction-Associated Steatotic Liver Disease (MASLD), there is little evidence of differences in BC in patients with MASLD regarding body mass index (BMI). The aim of this study was to determine differences in BC in terms of BMI and metabolic comorbidities in patients with MASLD.

**Materials and methods:**

It is a cross-sectional study with patients who attended the check-up unit. Liver steatosis was evaluated by controlled attenuation parameter, and patients were classified into five groups according to BMI, presence of MASLD, and metabolic characteristics: <25 kg/m^2^ non-MASLD; <25 kg/m^2^-MASLD; Overweight-MASLD; Metabolically Healthy Obese (MHO)-MASLD; and Metabolically Unhealthy Obese (MUO)-MASLD. BC was assessed by bioelectrical impedance and a Bioimpedance Vectorial Analysis (BIVA) was carried out. Differences in BC were analyzed by a One-Way ANOVA test. Univariate and multivariate analyses were performed for factors associated with abnormal BC.

**Results:**

A total of 316 patients were included. 59% (*n* = 189) were male, with a mean age of 49 ± 10 years. Fat% significantly higher according to BMI was not different between BMI <25 kg/m^2^-MASLD and Overweight-MASLD groups. Skeletal muscle mass (SMM) was significantly lower in obesity groups with respect to overweight and normal weight groups (*p* < 0.05); however, no differences were observed in the post-hoc analysis. Extracellular Water/Intracellular Water ratio was significantly higher in the MHO-MASLD group and MUO-MASLD group compared with the BMI <25 kg/m^2^ non-MASLD group and with the BMI <25 kg/m^2^-MASLD group. Abnormal Waist Circumference (WC) and liver steatosis were independent factors associated with abnormal BC.

**Conclusion:**

BC in MASLD patients varies according to BMI increase; changes could be explained by loss of SMM and not necessarily by the presence of metabolic abnormalities. High WC and the presence of steatosis are independent factors associated with altered BC.

## 1 Introduction

Metabolic diseases have been related to the body composition (BC) pattern, which is defined as the combination of variables that describe an individual’s distribution of fat and/or muscle, quantifying *in vivo* the body components, the quantitative relationships between the components, and their quantitative changes related to influential factors (1).

It is well known that due to the increase in the prevalence of obesity and diabetes mellitus (DM), other metabolic diseases have also increased; one of the most important is Metabolic Dysfunction-Associated Steatotic Liver Disease (MASLD), which nowadays is the most important chronic liver disease and one of the major indication for liver transplant worldwide, with an estimated prevalence of 30% (2, 3).

The inflammatory factors are one of the most important players in the relationship between metabolic diseases and BC (4). In MASLD patients, changes in BC are related to insulin resistance (IR), increase of lipolysis, and fatty acids accumulation in liver tissue (5); moreover, alterations in BC have been associated with an increased risk of presence and progression of MASLD (6–8).

In MASLD patients, BC assessment is important since it is associated with hepatic fat percentage and progression of liver disease (9, 10). Altered BC, characterized by high-fat tissue and low muscle mass, has been related to functional performance and metabolic comorbidities in patients with MASLD, especially in those with DM and cardiovascular diseases, which also get worse with fat tissue increase (10).

Whereas the Dual Energy X-ray Absorptiometry (DEXA) is the reference method for BC assessment (11), the Bioelectrical Impedance Analysis (BIA) has shown good concordance with DEXA, and it has been proposed as a good method of BC assessment in obese patients in whom the physiological and hydration conditions could interfere with measurement reliability (5, 12, 13). BIA is a noninvasive and relatively available method for BC analysis based on measuring resistance (R) and reactance (Xc), which allows to determine the fat and muscle percentage and hydration state through bioelectrical impedance vector analysis (BIVA) (14, 15).

While the relationship between changes in BC and the presence of liver steatosis has been established, there is little evidence about the characteristics of BC in patients with MASLD and differences related to body mass index (BMI); therefore, the aim of this study was to determine differences in BC according to BMI and metabolic comorbidities in patients with MASLD.

## 2 Materials and methods

### 2.1 Study population

This prospective study was carried out at the Medica Sur Clinic & Foundation check-up unit from March 2023 to January 2024, including patients between 18 and 70 years old. Demographic variables, hereditary family history, and pathological personal history of chronic degenerative diseases were collected as part of the check-up evaluation. We excluded patients with previous diagnoses of other liver diseases, such as viral hepatitis (hepatitis B or C virus infection), autoimmune hepatitis, hereditary diseases, liver cirrhosis, and those with hepatotoxic drugs treatment; laboratory tests and medical history confirmed the absence of these criteria during the check-up. This study was conducted in accordance with the Declaration of Helsinki and approved by the Institutional Ethics Committee of Medica Sur (2021-EXT-638).

### 2.2 Anthropometric and biochemical metabolic assessment

Anthropometric parameters of waist circumference (WC), weight, and height were collected; BMI was calculated as weight (kg)/height (m)^2^, and overweight was determined as BMI ≥25 kg/m2. Laboratory studies included blood count, blood chemistry, lipid profile, and liver function tests taken from blood samples after fasting for at least 8–12 h. Metabolic syndrome criteria were defined according to the Adult Treatment Panel III (16). Patients with obesity were divided into Metabolically Healthy Obesity (MHO; BMI ≥30 kg/m^2^ and one metabolic syndrome criteria) and Metabolically Unhealthy Obesity (MUO; BMI ≥30 kg/m^2^ and ≥2 metabolic syndrome criteria) (17).

### 2.3 MASLD diagnosis

MASLD was determined according to the definition criteria (3). Hepatic steatosis (dB/m) and liver fibrosis (skPa) were determined by transient elastography (TE; FibroScan®, Echosens™, 502 Touch, Paris, France) with Controlled Attenuation Parameter (CAP), with fasting for at least 4 h. It was performed by a single expert operator, using M or XL probe according to the manufacturer’s instructions and following the reliability criteria (IQR-CAP <40 and IQR-kPa <30) Patients whose studies did not meet the reliability criteria were excluded, as well as patients with F4 liver fibrosis according to TE (≥12 kPa). Steatosis determination was established according to Sirli et al.’s cut-off, being steatosis ≥263 dB (18). Once TE confirmed steatosis, MASLD was diagnosed if the patient had at least one of the cardiometabolic criteria (BMI ≥25 kg/m^2^; WC >94 (M) and >88 (F), fasting glucose ≥100 mg/dl or HbA1c ≥5.7% or DM or DM treatment, blood pressure ≥ 130/85 mmHg or antihypertensive treatment, and HDL < 40 (M) and < 50 (F) or lipid-lowering treatment). Patients with significant alcohol consumption [>140 g (F) and > 210 g (M)] referred in the medical record of the check-up were excluded.

Patients were classified into five groups according to BMI, the presence of MASLD, and metabolic abnormalities: BMI <25 kg/m^2^ non-MASLD, BMI <25 kg/m^2^-MASLD, overweight-MASLD, MHO-MASLD, and MUO-MASLD.

### 2.4 Body composition assessment

BC was analyzed by BIA by recording R and Xc using a four-terminal, single-frequency impedance analyzer (model Quantum IV-BIA; RJL-System, Detroit, MI, USA). BIA was conducted according to manufacturer’s recommendations. BC components (phase angle (PA), mass and percentages of fat, skeletal muscle mass (SMM), total body water (TBW), intracellular water (ICW), and extracellular water (ECW)) were calculated using the manufacturer’s software using the Mexican Adults equation set. Additionally, the ECW/ICW ratio was calculated. Body fluid variation was assessed by BIVA, according to Piccoli et al. (19), with the RXc graphic method, which analyzes the R and Xc values adjusted by height. BIVA graphics were generated using the Mexican population references (20).

### 2.5 Statistical analysis

Data distribution was determined by the Kolmogorov–Smirnov test. Then, continuous variables are reported as median and standard deviation, whereas categorical variables are expressed as percentages and frequencies. Differences in BC components were analyzed by one-way ANOVA test with Bonferroni post-hoc. First, we analyzed BC differences among all groups and then only in MASLD groups. Bivariate and multivariate analyses were carried out in these patients to determine the independent factors related to abnormal BC, with a percentile 75 of ECW/ICW ratio (≥0.95), and BIVA analysis as reference. A *p*-value <0.05 was considered statistically significant. All statistical analyses were conducted using the statistics program SPSS v20 (IBM Corp. Released 2011. IBM SPSS Statistics for Windows, Version 20.0. Armonk, NY: IBM Corp.).

## 3 Results

A total of 316 patients were included: BMI <25 kg/m^2^ non-MASLD (*n* = 70), BMI <25 kg/m^2^-MASLD (*n* = 36), overweight-MASLD (*n* = 70), MHO-MASLD (*n* = 70), and MUO-MASLD (*n* = 70). 59.6% (*n* = 189) were male with a mean age of 49 ± 10 years; at the time of evaluation, 6.9% (*n* = 22) had a known diagnosis of DM and 16.7% (*n* = 53) had a known diagnosis of high blood pressure. Concerning metabolic risks, a decreased High-density Lipoprotein (HDL) level was the most prevalent (38.2%, *n* = 121), followed by abnormal triglycerides (34.1%, *n* = 108), and glucose impairments (28.1%, *n* = 89). The mean of dB/m was 287.9 ± 55.2; meanwhile, the mean of kPa was 3.7 ± 0.8. 1.2% (*n* = 4) of patients have significant fibrosis (8.0–11.9 kPa). General characteristics of patients are presented in Table 1.

**Table 1:** General characteristics of patients.

Characteristic	All patients (*n* = 316)	BMI <25 kg/m^2^ non-MASLD (*n* = 70)	BMI <25 kg/m^2^-MASLD (*n* = 36)	Overweight-MASLD (*n* = 70)	MHO-MASLD (*n* = 70)	MUO-MASLD (*n* = 70)	*p* ^*^
	*n* (%), μ ± SD	*n* (%), μ ± SD	*n* (%), μ ± SD	*n* (%), μ ± SD	*n* (%), μ ± SD	*n* (%), μ ± SD	
Male	59.6 (189)	43.7 (31)	55.6 (20)	62.9 (44)	64.3 (45)	70 (49)	0.01
Age (years)	49.2 ± 10.5	49.1 ± 12.6	50.8 ± 9.3	50.2 ± 10	48.3 ± 9.9	48.5 ± 10.1	0.68
DM	6.9 (22)	1.4 (1)	-	11.4 (8)	7.1 (5)	11.4 (8)	0.03
Dyslipidemia	18.6 (59)	9.9 (7)	19.4 (7)	34.3 (24)	11.4 (8)	18.6 (13)	0.002
HT	16.7 (53)	1.4 (1)	5.6 (2)	24.3 (17)	22.9 (16)	24.3 (17)	≤0.001
BMI kg/m^2^	28.4 ± 5.0	22.3 ± 1.8	23.7 ± 1.0	27.8 ± 1.3	32.6 ± 2.3	33.4 ± 3.2	≤0.001
WC cm	98.2 ± 15.0	81.2 ± 12.6	88.7 ± 6.7	97.2 ± 7.6	109.3 ± 9.5	110.3 ± 9.2	≤0.001
SBP mmHg	118.9 ± 16.7	107.7 ± 12.8	114.9 ± 16.9	116.8 ± 14.8	122.2 ± 14.6	131.4 ± 14.6	≤0.001
DBP mmHg	76.8 ± 10.8	69.0 ± 8.4	75.8 ± 9.5	75.1 ± 9.9	79.4 ± 10.1	84.4 ± 9.2	≤0.001
Fasting glucose mg/dl	95.9 ± 18.8	88.7 ± 12.5	95.6 ± 10.8	96.3 ± 13.9	91.1 ± 8.6	107.7 ± 30.4	≤0.001
Triglycerides mg/dl	143.0 ± 90.9	88.0 ± 40.1	178.8 ± 132.2	144.4 ± 68.7	113.9 ± 39.3	208.4 ± 110.1	≤0.001
HDL mg/dl	48.8 ± 13.7	57.0 ± 13.9	50.5 ± 14.3	49.6 ± 13.0	49.2 ± 11.1	38.3 ± 9.3	≤0.001
HbA1C %	5.4 ± 0.7	5.2 ± 0.5	5.4 ± 0.4	5.3 ± 0.8	5.5 ± 0.3	5.8 ± 1.0	≤0.001
CRP mg/L	3.0 ± 3.9	1.7 ± 3.6	1.9 ± 2.3	2.7 ± 2.5	4.3 ± 5.4	4.0 ± 3.7	≤0.001
dB/m	287.9 ± 55.2	206.0 ± 25.7	287.9 ± 25.2	306.8 ± 31.2	312.3 ± 32.4	327.9 ± 38.3	≤0.001
skPa	4.1 ± 1.0	3.7 ± 0.8	4.3 ± 1.4	3.9 ± 0.7	4.1 ± 1.0	4.6 ± 1.1	≤0.001

BMI, Body Mass Index; MASLD, Metabolic Dysfunction-Associated Steatotic Liver Disease; MHO, Metabolically Healthy Obesity; MUO, Metabolically Unhealthy Obesity; DM, Diabetes Mellitus; HT, Hypertension; WC, Waist Circumference; SBP, Systolic Blood Pressure; DBP, Diastolic Blood Pressure; HDL, High-Density Lipoprotein; HbA1C, Glycosylated hemoglobin; CRP, C-Reactive Protein.

* *p-value represents the comparison among groups.*

**Figure 1:**
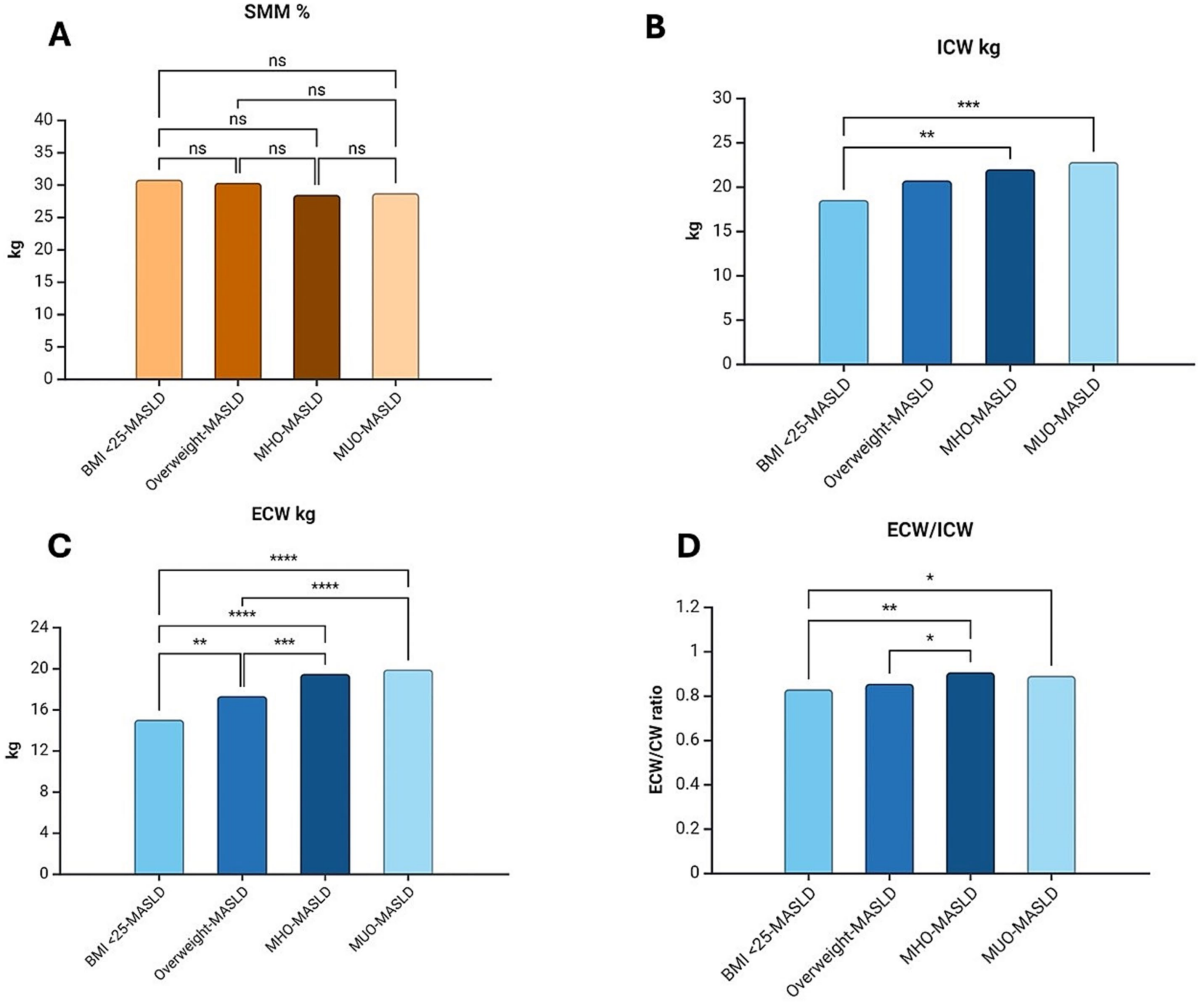
Comparison of body composition components among groups. (A) SMM showed differences in the comparison of all groups; however, although SMM showed lower values, differences were not observed in a *post hoc* analysis. (B) ICW (kg) was significantly higher when the BMI < 25-MASLD group was compared with obesity-MASLD groups. (C) ECW (kg) did not show significant differences in obesity groups. (D) ECW/ICW ratio shows differences among the BMI < 25-MASLD group and the obesity groups and between overweight and MHO-MASDL groups. SMM, Skeletal Muscle Mass; BMI, Body Mass Index; MASLD, Metabolic Dysfunction-Associated Steatotic Liver Disease; MHO, Metabolically Healthy Obesity; MUO, Metabolically Unhealthy Obesity; ICW, Intracellular Water; ECW, Extracellular Water; ns non-significative; *; **; ***; **** *p* < 0.05. Created with BioRender.com

Regarding the analysis of BC differences among all groups (Table 2), R and Xc show significant differences (*p* ≤ 0.0001), and PA did not show differences among groups. As expected, Fat% was significantly increased in terms of BMI (*p* ≤ 0.0001); however, in post-hoc analysis, Fat% was not different between the BMI <25 kg/m^2^-MASLD (34.9 ± 6.7%) and Overweight-MASLD (36.5 ± 6.3%) groups. SMM% was significantly lower in obesity groups with respect to overweight and normal weight groups (*p* < 0.05); no significant differences were observed among overweight and normal weight groups. Despite the differences among all groups (*p* ≤ 0.001), no significant differences were observed between BMI <25 kg/m^2^ groups with (32.4 ± 6.2 kg) or without MASLD (33.5 ± 5.9 kg), and Overweight MASLD (38.1 ± 7.2 kg) compared to MHO-MASLD group (41.3 ± 8.4 kg) regarding water-related components. ECW was significantly higher according to BMI increase; however, no differences were observed according to metabolic health or unhealth in obesity groups. ECW/ICW ratio was significantly higher in the MHO-MASLD group and MUO-MASLD group compared with the BMI <25 kg/m^2^ non-MASLD group (*p* = 0.001 and *p* = 0.02, respectively), and with the BMI <25 kg/m^2^-MASLD group (*p* = 0.001 and *p* = 0.01, respectively; Figure 1D).

**Table 2:** Differences in body composition components among groups.

Component	BMI <25 kg/m^2^ non-MASLD (*n* = 70)	BMI <25 kg/m^2^-MASLD (*n* = 36)	Overweight-MASLD (*n* = 70)	MHO-MASLD (*n* = 70)	MUO-MASLD (*n* = 70)	*p* ^*^
	μ ± SD, %	μ ± SD, %	μ ± SD, %	μ ± SD, %	μ ± SD, %	
Resistance Ω	598.3 ± 67.9	597.4 ± 77.4	525.4 ± 63.6	501.8 ± 69.3	482.9 ± 535.3	**≤0.001**
Reactance Ω	65.3 ± 8.7	66.4 ± 7.3	59.7 ± 6.5	57.5 ± 7.8	55.5 ± 6.8	**≤0.001**
PA °	6.2 ± 0.9	6.4 ± 0.8	6.5 ± 0.7	6.5 ± 0.7	6.6 ± 0.7	0.09
Fat kg	19.6 ± 5.2	23.7 ± 4.9	28.6 ± 6.4	38.7 ± 12.3	38.7 ± 7.4	**≤0.001**
Fat %	31.5 ± 7.8	34.9 ± 6.7	36.5 ± 6.3	40.6 ± 8.1	40.3 ± 6.0	**≤0.001**
TBW kg	32.4 ± 6.2	33.5 ± 5.9	38.1 ± 7.2	41.3 ± 8.4	42.6 ± 8.1	**≤0.001**
TBW %	51.6 ± 5.1	48.7 ± 4.8	47.5 ± 5.3	43.6 ± 6.7	44.0 ± 4.6	**≤0.001**
ICW kg	17.9 ± 4.2	18.5 ± 4.0	20.7 ± 4.6	21.9 ± 5.2	22.9 ± 5.2	**≤0.001**
ICW %	28.2 ± 4.1	26.8 ± 4.0	25.8 ± 4.1	23.2 ± 4.7	23.5 ± 3.4	**≤0.001**
ECW kg	14.5 ± 2.2	14.5 ± 3.3	17.3 ± 2.7	19.4 ± 3.6	19.8 ± 3.2	**≤0.001**
ECW %	23.0 ± 2.1	21.8 ± 1.3	21.5 ± 1.5	20.4 ± 2.2	20.5 ± 1.3	**≤0.001**
SMM kg	20.3 ± 5.3	21.1 ± 4.8	24.3 ± 5.1	27.0 ± 6.4	27.7 ± 6.4	**≤0.001**
SMM %	31.9 ± 5.7	30.7 ± 5.3	30.2 ± 4.4	28.4 ± 5.0	28.6 ± 4.2	**≤0.001**
ECW/ICW	0.82 ± 0.10	0.80 ± 0.17	0.85 ± 0.10	0.90 ± 0.11	0.89 ± 0.11	**≤0.001**

BMI, Body Mass Index; MASLD, Metabolic Dysfunction-Associated Steatotic Liver Disease; MHO, Metabolically Healthy Obesity; MUO, Metabolically Unhealthy Obesity; PA, Phase Angle; TBW, Total Body Water; ICW, Intracellular Water; ECW, Extracellular Water; SMM, Skeletal Muscle Mass.

* *p-value represents the comparison among groups. Bold values represents p-values <0.05.*

Once again, only in MASLD groups (*n* = 246) all components showed differences in the One-way ANOVA test, except PA, where differences in BC were analyzed. Fat% was higher according to BMI; however, there was no difference between the BMI <25 kg/m^2^-MASLD group and the Overweight-MASLD group (34.9 ± 6.7% vs. 36.5 ± 6.3%, *p* = 1.00), nor between the MHO-MASLD group and the MUO-MASLD group (40.6 ± 8.1 vs. 40.3 ± 6.0%, *p* = 1.00) in post-hoc analysis. SMM% was significantly different among groups (*p* = 0.01), being higher in BMI <25 kg/m^2^-MASLD group and Overweight-MASLD group than in Obesity Groups (30% vs. 28%), but no significant differences were observed in post-hoc analysis (Figure 1A).

ICW was significantly higher only among the BMI <25 kg/m^2^-MASLD and MHO-MASLD (18.5 ± 4.0 kg vs. 21.9 ± 5.2 kg, *p* = 0.003) and MUO-MASLD groups (18.5 ± 4.0 kg vs. 22.8 ± 5.0, *p* = 0.0002; Figure 1B). Instead, ECW was significantly different among all groups, increasing in terms of BMI, but once again, without difference in obesity groups (Figure 1C). When the ECW/ICW ratio was analyzed, we observed an increase according to BMI; however, the BMI <25 kg/m^2^-MASLD group only showed significant differences with MHO-MASLD and MUO-MASLD groups (0.80 ± 0.1 vs. 0.90 ± 0.1, *p* = 0.005, and 0.80 ± 0.1 vs. 0.89, *p* = 0.04, respectively), whereas the Overweight-MASLD group only showed differences with the MHO-MASLD group (0.85 ± 0.1 vs. 0.90 ± 0.1, *p* = 0.04; Figure 1D).

**Figure 2:**
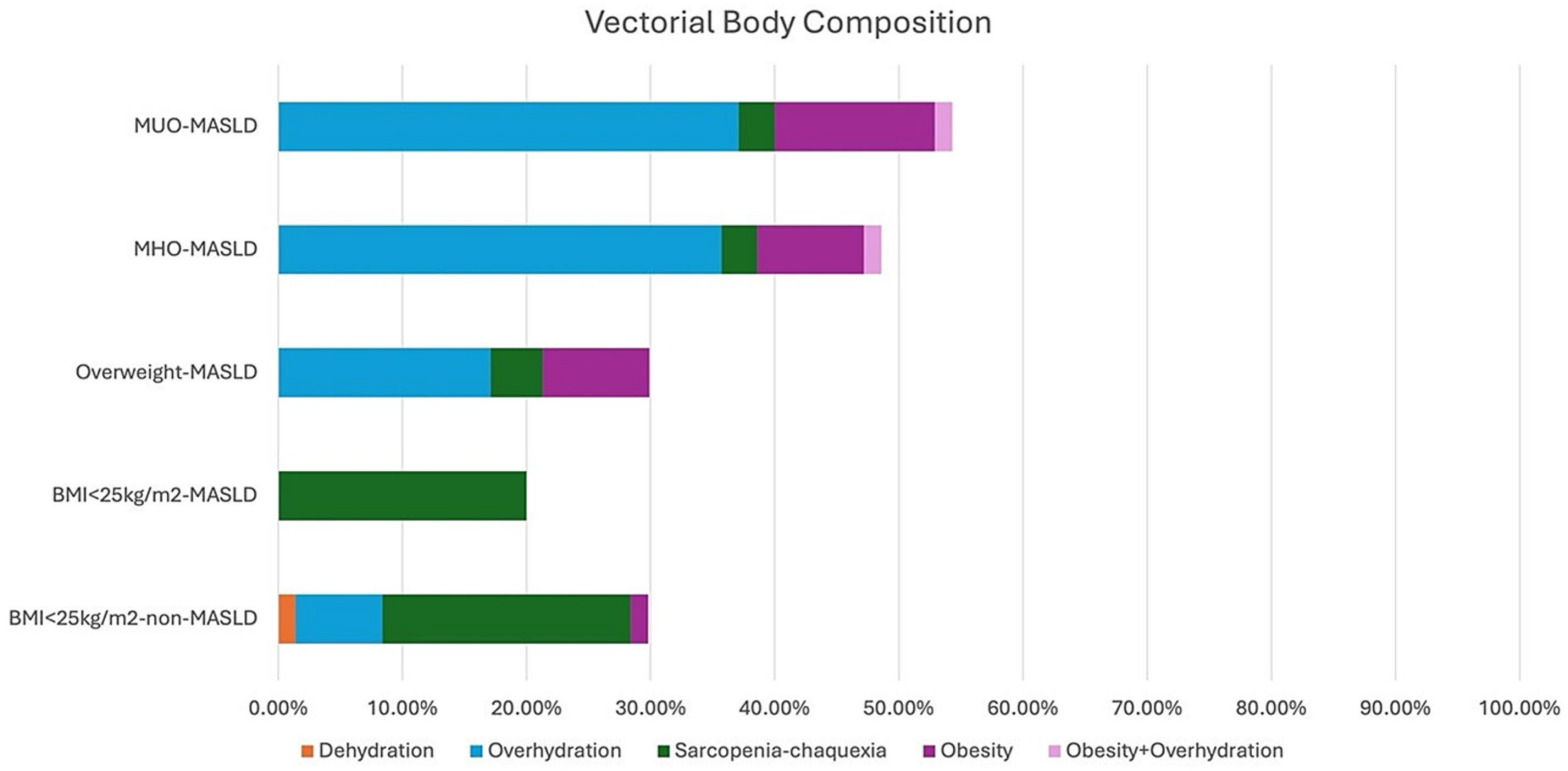
Bioimpedance Vectorial Analysis in each group. Graphic representation of body composition in each group, for male and female patients, according to tolerance ellipses for the Mexican population. BMI, Body Mass Index; MASLD, Metabolic dysfunction-associated Steatotic Liver Disease; MHO, Metabolically Healthy Obesity; MUO, Metabolically Unhealthy Obesity; Xc, Reactance; H, Height.

**Figure 3:**
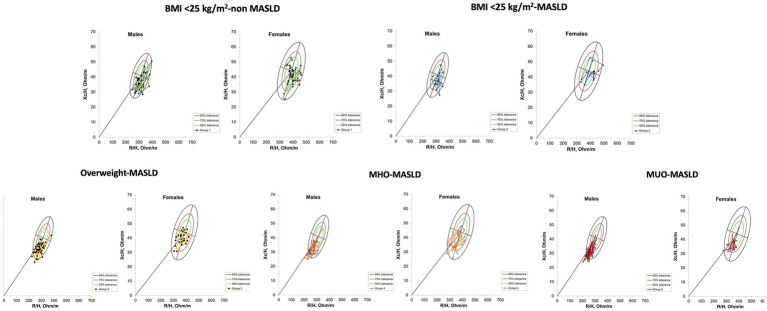
Classification of patients according to Bioimpedance Vectorial Analysis. BMI, Body Mass Index; MASLD, Metabolic Dysfunction-Associated Steatotic Liver Disease; MHO, Metabolically Healthy Obesity; MUO, Metabolically Unhealthy Obesity.

The differences in water components were confirmed with the BIVA qualitative analysis. According to the RXc point graphic and tolerance ellipses, with the increase of BMI, the points were situated in vectors that represent more fluids but not necessarily in those that represent fewer lean tissues (21, 22) (Figure 2). Regarding the BIVA tissue classification, normal tissue was majorly prevalent in the BMI <25 kg/m^2^ non-MASLD (70.4%, *n* = 50/70) group, BMI <25 kg/m^2^-MASLD (83.3%, *n* = 30/36) group, and Overweight-MASDL (70%, *n* = 49/70) group; however, it was decreased in the MHO-MASLD (51.4%, *n* = 36/70) group and MUO-MASLD (45.7%, *n* = 32/70) group. The prevalence of sarcopenia-cachexia tissue was higher in BMI <25 kg/m^2^ groups (20%), and lower in Overweight-MASLD (4.2%, *n* = 3) group, MHO-MASLD group, and MUO-MASLD group (2.9%, *n* = 2, both). Conversely, overhydration was higher in Overweight-MASLD (17.1%, *n* = 12) group, MHO-MASLD (35.7%, *n* = 25) group, and MUO-MASLD (37.1%, *n* = 26) group. In the BMI <25 kg/m^2^ non-MASLD group, only 7% (*n* = 5) presented overhydration, and it was not present in the BMI <25 kg/m^2^-MASLD group (Figure 3).

Factors associated with abnormal BC were analyzed, according to the ECW/ICW ratio and the BIVA tissue classification. In all patients (*n* = 316), female sex, abnormal WC, abnormal HDL levels, and the presence of liver steatosis were independent factors associated with abnormal BC, according to ECW/ICW ratio (Table 3); as for BIVA, female sex [OR 3.3 CI95% (1.9–5.5), *p* ≤ 0.001] and abnormal WC [OR 2.2 CI95% (1.3–3.9), *p* ≤ 0.001] were independently associated with abnormal BC (Table 3). We performed the bivariate and multivariate analyses adjusting by sex, and then the abnormal WC and the presence of liver steatosis maintained the independent association with abnormal BC, according to ECW/ICW ratio (Table 4); as for BIVA, only abnormal WC [OR 2.7 CI95% (1.4–5.2), *p* = 0.003] was independently associated with abnormal BC (Table 4). When these analyses were carried out, only in MASLD groups, female sex, and abnormal WC were independent factors associated with abnormal BC in both criteria (data not shown); in adjusted analysis by sex, abnormal WC maintained the independent association with BIVA [OR 4.3 CI 95% (1.8–10.1), *p* = 0.001] as reference, but abnormal WC only showed association in bivariate analysis [OR 3.6 CI 95% (1.8–7.3), *p* = 0.001] with ECW/ICW ratio as reference.

**Table 3:** Univariate and multivariate analysis for abnormal body composition according to ECW/ICW > 0.95 and BIVA.

Factor	Univariate		Multivariate	
	OR (CI 95%)	*p*	OR (CI 95%)	*p*
ECW/ICW >0.95				
Female	14.2 (7.1–28.4)	**≤0.001**	78.0 (30.7–198.0)	**≤0.001**
WC	2.2 (1.4–3.6)	**≤0.001**	2.8 (1.0–7.3)	**0.03**
Abnormal HDL	1.5 (1.1–2.2)	**0.018**	2.3 (1.1–5.1)	**0.02**
HbA1c ≥5.6%	1.6 (1.0–2.1)	0.063		
Liver steatosis	2.1 (1.1–3.9)	**0.006**	4.0 (1.3–11.9)	**0.01**
BIVA				
Female	2.2 (1.5–3.1)	**≤0.001**	3.3 (1.9–5.5)	**p ≤ 0.001**
WC	1.7 (1.2–2.4)	**0.001**	2.2 (1.3–3.9)	**p ≤ 0.001**
HT history	1.3 (0.9–1.8)	0.08		
Abnormal AT	1.5 (1.1–2.0)	**0.02**		

ECW/ICW, Extracellular Water–Intracellular Water ratio; BIVA, Bioimpedance Vectorial Analysis; WC, Waist Circumference > 88 cm in women and > 102 in men; HDL, High-Density Lipoprotein < 50 in women and < 40 in men; HbA1c, Glycosylated Hemoglobin; HT, Hypertension. Bold values represents *p*-values <0.05.

**Table 4:** Univariate and multivariate analysis for abnormal body composition according to ECW/ICW > 0.95 and BIVA, adjusted by sex.

Factor	Univariate		Multivariate	
	OR (CI 95%)	*p*	OR (CI 95%)	*p*
ECW/ICW >0.95				
WC	2.8 (1.8–4.3)	**≤0.001**	4.5 (1.5–12.9)	**0.005**
Abnormal BP	1.5 (1.2–2.0)	**0.050**		
Abnormal HDL	1.4 (1.1–1.9)	**0.006**		
HbA1c >5.6%	1.3 (1.0–1.7)	0.069		
Liver steatosis (>263 dB/m)	3.4 (1.9–6.1)	**≤0.001**	3.9 (1.2–12.4)	**0.017**
BIVA				
WC	1.9 (1.3–2.8)	**≤0.001**	2.7 (1.4–5.2)	**0.003**
HT history	1.3 (0.9–1.8)	0.09		
Abnormal HDL	1.4 (1.0–1.8)	**0.02**		

ECW/ICW, Extracellular Water–Intracellular Water ratio; BIVA, Bioimpedance Vectorial Analysis; WC, Waist Circumference > 88 cm in women and > 102 in men; BP, Blood pressure; HDL, High-Density Lipoprotein < 50 in women and < 40 in men; HbA1c, Glycosylated Hemoglobin; HT, Hypertension. Bold values represents *p*-values <0.05.

## 4 Discussion

The evidence of alterations in body composition in the MASLD scenario is scarce. In our study, changes in BC in patients with MASLD were observed, with significant differences compared to healthy patients (BMI < 25 kg/m^2^ and non-MASLD). As expected, Fat%, TBW, and ECW/ICW ratio were increased according to BMI increase, and conversely, SMM was decreased. However, significant differences were not observed among all groups and in MASLD groups in post-hoc analysis.

Fat accumulation is now considered a major risk factor for mortality, independent of obesity (23). A significant increase in Fat% has been observed in patients with MASLD and BMI < 25 kg/m^2^; this has been observed in the United States population by Mainous III et al. (24) and in the Rotterdam cohort (OR 1.77, *p* ≤ 0.05) (25). In our population, we observed a significant difference in the increase of Fat% in patients with BMI <25 kg/m^2^, being higher in those with MASLD. The prevalence of MASLD in patients with BMI <25 kg/m^2^ is relatively low; in our population, we previously reported a prevalence of 7.9% (26), even though the BMI < 25 kg/m^2^-MASLD group is smaller than the other groups. One of the strengths of our study is the inclusion and comparison of this group of patients with other MASLD phenotypes, taking their low prevalence into account. BC assessment could be an early detection tool in these patients in whom MASLD is not an initial clinical suspicion. Another strength of this study is the BIVA analysis, which is the qualitative point of view of BC. As far as we know, it has not been evaluated in patients with MASLD. According to our results, changes observed in BC are consistent with BIVA in overhydration and lean mass tissue terms, according to the tolerance ellipses and BIVA tissue classification (Figures 2, 3).

Abdominal fat accumulation seems to be a better indicator of MASLD than BMI or the presence of obesity (27). In our study, we observed a significant increase in Fat% in both BMI <25 kg/m^2^ and overweight/obese patients. On the other hand, WC was an independent factor associated with altered BC in all patients and also when only MASLD patients were analyzed, without differences among the number of comorbidities in obese patients.

Sarcopenia increases in MASLD and is considered a progression factor independent of obesity and IR. Muscle strength was not evaluated in our study, so we cannot use the sarcopenic obesity concept (decreased muscle mass, increased fat, and decreased muscle strength) (28). We refer to myopenia instead, which exclusively refers to low muscle mass (23); in obese patients, it will be *myopenic obesity.*

In patients with MASLD, a decrease in SMM has been associated with BMI, Fat Mass Index, and WC, with significant differences regarding sex, majorly attributed to hormones. Onishi et al. (10) evaluated the associated factors to SMM decrease in patients with MASLD, finding that BMI, Fat-free mass Index, and WC were independent associated factors. However, the study was conducted in an Asian population with a different BMI cut-off to determine overweight. Despite this, our results confirm that SMM is significantly lower in patients with MASLD according to BMI increase; however, despite detecting a trend, no significant differences were observed in terms of BMI classification or the presence of comorbidities in obese patients in a post-hoc analysis. Statistical significance could be lost since our study universe corresponds to an open-apparently healthy population that attended a check-up unit with an overall mean age (49.2 ± 10.5) and stage of liver steatosis (287.9 ± 55.2) in which significant muscle loss is not expected. However, this observed trend is clinically significant for early recognition of decreased SMM.

As for water measurements, we observed a significant decrease in the TBW percentage, according to BMI increase (Table 2). TBW percentage has been observed to reflect higher levels of adiposity, and this could affect the reliability of measurements of fat-free mass. However, this could produce a clinical underestimation of obesity if only TBW is considered for body composition assessment (20). Therefore, the evaluation of TBW components is a more reliable measurement, especially in obese patients, since one of the characteristics of obesity is an alteration in fluid regulation; changes in ECW and ICW have been attributed to the high proportion of ECW in adipose tissue, the relationship of ECW with chronic inflammation (29), obesity-related edema, and hormonal responses to fat tissue, leading to a primary deficiency in hemodynamic fluid regulation that could not be reversible in morbid obesity (30, 31).

The increase in water components of BC at the expense of increased fat could be the explanation for the difficulty of muscle mass recovery, even in lean patients; moreover, this fluid alteration seems to persist after weight loss becomes irreversible (30, 32).

Although it is interesting to highlight that the ECW/ICW ratio has demonstrated to be an overload water marker and, even more, a mortality marker in populations different than ours and in cardiovascular risk populations (15, 33–37), there is no evidence of this ratio in MASLD patients. However, different studies (33, 36, 37) show that this ratio could be an early marker of muscle mass and function loss. In our study, this ratio was higher in patients with BMI >25 kg/m^2^; therefore, if we evaluate it with SMM percentage, even if no statistical difference was observed, it could be considered an early marker of sarcopenia.

Regarding PA, Chen et al. (38) observed that it is lower in patients with MASLD compared to non-MASLD patients. When the analysis was adjusted by BMI, sex, and comorbidities, PA was associated with MASLD risk; however, this association was not observed in patients with BMI >30 kg/m^2^. The authors concluded that PA could be an indicator in MASLD management limited to overweight patients.

Abnormal BC has been established as a risk factor and as an indicator for the presence of liver steatosis (8, 39). According to our results, liver steatosis is an independent factor associated with altered BC when it is defined by the ECW/ICW ratio in multivariate analysis. Therefore, changes in BC seem to be one more factor affected by MASLD development.

From the anthropometric point of view, the assessment of MASLD patients would need deeper indicators than BMI, including BC analysis, which seems to be a tool for patients’ diagnosis, classification, muscle mass measurement, and follow-up. Improvement of BC has been related to a decrease in liver fat content in patients with MASLD (40). Currently, there is insufficient evidence to assess whether newest treatments that have demonstrated to reverse steatosis or fibrosis also impact BC. Although weight loss is the cornerstone of MASLD treatment, it is important to evaluate whether treatment schemes could have a “negative” impact on BC, especially in those patients with increased Fat% and decreased SMM in whom weight loss without improvement or maintenance of SMM could remain a risk for metabolic and cardiovascular mortality, despite weight loss.

## 5 Conclusion

BC in MASLD patients varies according to BMI increase; changes could be explained by loss of SMM and not necessarily by the presence of metabolic abnormalities. High WC and the presence of steatosis are independent factors associated with altered BC.

## Data Availability

The raw data supporting the conclusions of this article will be made available by the authors, without undue reservation.
